# The association between the Kidney Donor Profile Index and one-year outcomes in Brazilian kidney transplant recipients of standard criteria donors

**DOI:** 10.1590/2175-8239-JBN-2024-0219en

**Published:** 2025-07-21

**Authors:** Ana Paula Aquino de Morais, Renato Demarchi Foresto, Maria Amélia Aguiar Hazin, Bianca Cristina Cassão, Helio Tedesco-Silva, José Medina Pestana, Lúcio Requião-Moura

**Affiliations:** 1 Universidade Federal de São Paulo, Departamento de Medicina, São Paulo, SP, Brazil.; 2 Fundação Oswaldo Ramos, Hospital do Rim, São Paulo, SP, Brazil.

**Keywords:** Kidney Transplantation, Tissue Donors, Brain Death, Graft Survival, Glomerular Filtration Rate

## Abstract

**Introduction:**

The Kidney Donor Profile Index (KDPI) has not been previously validated in Brazil, thus this study aimed to investigate the association between the index and one-year outcomes in kidney transplant recipients (KTRs) of standard criteria donors (SCD).

**Methods:**

Retrospective cohort analysis including 1,943 KTRs who received kidneys from SCD between 2013 and 2017. The primary outcome was composed of death, graft loss, and 1-yr-graft function <30 mL/min/1.73m^2^ (eGFR, CKD-EPI). Logistic regression identified variables associated with the primary outcome, while 1-yr eGFR across KDPI strata was compared using the Kruskal-Wallis test, adjusted with the Bonferroni test.

**Results:**

Donors were 41.0 years old, 24.9% had hypertension, 47.3% died due to cerebrovascular injury, and 48.3% had the final creatinine >1.5 mg/dL; the median of KDPI was 52%. The primary outcome occurred in 14.4% of the cases, which was associated with longer dialysis duration before transplantation (p = 0.04), CMV-related events (p = 0.03), acute rejection (p < 0.001), and KDPI strata. Compared to the 1-35% KDPI stratum, the RRs for the primary outcome were significantly higher in higher KDPI strata: 1.62 (p = 0.03) for >35–50%, 2.28 (p < 0.001) for >50–85%, and 2.23 (p = 0.01) for >85%. The 1-yr eGFR was also significantly lower in KTRs with donors in higher KDPI strata (p < 0.001).

**Conclusion:**

The KDPI was independently associated with the primary outcome composed of death, graft loss, and 1-yr eGFR <30 mL/min/1.73 m^2^ in recipients of SCD donors. Despite not being previously validated for Brazilian donors, the KDPI was also significantly associated with 1-yr eGFR, delayed graft function, and acute rejection in those recipients.

## Introduction

End-stage chronic kidney disease (CKD) is a pressing public health concern^1,2^, and kidney transplantation is the best long-term treatment option for patients in this condition^3,4^. However, even in countries with large- scale transplant programs like Brazil, there is still a shortage of organ donors^5^. This scarcity of donors has prompted a reevaluation of donor acceptance criteria in recent decades, leading to the inclusion of risk classifications based on donor characteristics in long-term outcome assessments, affecting the decision to use organs that might otherwise be discarded^6,7^. Therefore, over the past two decades, deceased kidney donors have been categorized into standard or expanded criteria donors (SCD/ECD) based on the United Network for Organ Sharing (UNOS) criteria. Donor characteristics have been identified, which, either individually or in combination, represent a 70% increased risk of long-term graft loss, distinguishing between ECD and SCD^8^.

Although the UNOS criteria offer valuable guidance to transplant teams, they do have limitations, such as not considering diabetes as a relevant prognostic factor, underestimating the risk associated with young donors with multiple comorbidities, and overestimating the risk of previously healthy elderly donors. To address these limitations and enhance the precision of risk assessment, Rao et al. proposed the Kidney Donor Risk Index (KDRI), which incorporates a broader range of variables into the prediction formula^9^. The KDRI results are subsequently ranked on a percentile scale known as the Kidney Donor Profile Index (KDPI). This approach provides transplant teams and patients with a more detailed assessment of organ quality, facilitating multiple donor comparisons for more informed decision making^10^. Additionally, although the KDRI/KDPI was developed as an outcome prediction tool, its discriminatory power is moderate, and best in cases with extreme risk levels^9^.

While the KDPI has been validated in a few countries^11,12,13,14,15^, it has not yet been validated in Brazil. Despite initial investigations into the overlap of the two scoring systems during the creation of the KDRI, further research is needed in populations from different countries. Notably, in the cohort used to establish the UNOS criteria, approximately 15% of the donors met the ECD classification^8^. However, in the cohort from which the KDRI was derived, 32.8% of donors falling below the 85^th^ percentile was categorized as ECD, and 4.6% exceeding the 85^th^ percentile was classified as SCD^9^. This divergence is crucial given the anticipated improvements in outcomes for recipients of SCD organs.

Considering the overlap between the KDPI and UNOS criteria and that in our previous analysis, all donors with KDPI <50% were SCD, but still 72.8% and 9.8% were classified as SCD in the KDPI strata 51-85% and >85%, respectively,^16^ we hypothesized that even recipients of standard kidneys but with higher KDPI are at risk for poor outcomes after transplantation. Thus, this study aimed to investigate the association between KDPI and one-year outcomes in kidney transplant recipients (KTRs) of SCD in a population for which the index has not been previously validated, as one of the steps to validate the KDPI for Brazilian donors.

## Methods

### Study Design and Population

This was a retrospective single-center cohort study carried out at Hospital do Rim, São Paulo, SP, Brazil. Adult KTRs transplanted between January 2013 and December 2017 and who received a kidney graft from SCD were enrolled and followed-up for 12 months after transplantation. The study was conducted following the Declaration of Helsinki and approved by the local Ethics Committee (identification number CAEE 10021419.0.0000.5505 and approval number 3.274.985). The informed consent form was waived. The authors had access to electronic medical reports and institutional databases after the Ethical Committee approval. Data collection and curation were performed between May 2^nd^, 2019 and January 21^st^, 2021. The dataset was anonymized and deidentified. For study conception and presentation, the authors used as reference the STROBE and RECORD check list, as previously published^17^.

The eligible participants were KTRs who underwent kidney transplant from deceased donors under any immunosuppression maintenance regime. From 2013 and 2017, 3,161 kidney transplants from deceased donors were performed at Hospital do Rim. Patients who received kidney transplant combined with another solid organ (101 kidney-pancreas and 1 kidney-liver) and those who received a kidney graft from ECD (n = 1,116) were excluded. Thus, 1,943 individuals were included in the present analysis. As the study aimed to evaluate outcomes in the recipients, when a single donor provided both kidneys for the recipients included in the cohort, the donor’s characteristics were considered separately for each corresponding recipient.

### Immunosuppression and Definitions

Only patients with donor specific antibody (DSA) at HLA loci A and B and DR with mean fluorescence intensity (MFI) below 1,500 received a kidney transplant. Those with DSA at loci HLA A and B and DR with MFI between 300 and 1,500 received a kidney transplant and an additional 1 mg/kg of rabbit antithymocyte globulin (r-ATG) after the standard induction dose. According to the local practices, all patients receive induction therapy with a single dose of 3.0 mg/kg thymoglobulin^18^. The maintenance immunosuppression regimen consists of tacrolimus, prednisone, and a third drug defined by the recipients’ immunological risk, stratified by the calculated panel reactive antigen (cPRA): mycophenolate acid (MPA) for recipients with a cPRA >50% and azathioprine or mammalian target of rapamycin inhibitors (mTORi) for those with cPRA <50%.

Patients with PRA ≥ 50% receive MPA 720 mg BID. Tacrolimus is initially prescribed at a 0.1 mg/kg dose BID, and further adjusted to maintain a trough level between 5 and 10 ng/mL. Recipients of SCD kidneys with PRA<50% receive azathioprine 2 mg/kg QID or mTORi, according to other clinical characteristics of the recipient. Tacrolimus is initially prescribed at a 0.1 mg/kg dose BID, and further adjusted to maintain a trough level between 8 and 12 ng/mL for those receiving azathioprine. For those receiving mTORi, tacrolimus is initially prescribed at a 0.05 mg/kg dose BID, and further adjusted to maintain a trough level between 3 and 5 ng/mL. All patients receive 0.5 mg/kg prednisone (maximum dose of 30 mg), tapered weekly until 5 mg on day 30. The KDPI score was not used as a variable for choosing immunosuppression in our institution. According to the local team decision, other characteristics of donors or recipients can be considered when selecting the third drug.

The KDPI score is not considered for the decision on the maintenance immunosuppression regimen. All patients receive prophylaxis for *Pneumocystis jirovecii* with sulfamethoxazole-trimethoprim. The strategy to reduce the risk of events related to cytomegalovirus (CMV) is the preemptive treatment. According to local practices, the CMV-related event occurs when patients under preemptive treatment need antiviral strategy. Details about the local strategy for preemptive strategy were previously published^19^. In summary, CMV viremia was monitored between 2013 and February 2017 using pp65 antigenemia for preemptive treatment. After February 2017, the polymerase chain reaction (PCR) test was incorporated, but during the study period, the decision to initiate antiviral treatment was based on antigenemia results. The local preemptive strategy recommends antiviral treatment when 10 or more positive cells are detected in asymptomatic patients or any patient showing symptoms of CMV infection, regardless of the number of positive cells. Treatment consists of intravenous 5 mg/kg ganciclovir twice daily adjusted for renal function. Monitoring was performed weekly during treatment. A CMV-related event was defined as any instance where antiviral treatment was required, according to the local criteria.

Since 2013, the center has routinely calculated the KDPI/KDRI for each deceased donor, with the data being included in medical reports and institutional datasets. For the present study, all data were extracted from these records and rechecked. In cases where the KDRI/KDPI were not available (n = 555, 28.6%), they were retrospectively calculated using US data corresponding to the period of data collection and curation. Between 2013 and 2017, hepatitis C (HCV)- positive donors were not eligible for allocation in Brazil. Finally, the Brazilian allocation system does not allow the utilization of organs after cardiac death (DCD). Therefore, all donors in the study were HCV-negative and had brain death.

Delayed graft function (DGF) occurred when patients required dialysis in the first week following the transplantation. Acute rejection (AR) was defined by biopsy according to the Banff criteria^20^ or by presumed episodes, defined by recipients presenting a successfully treated episode of acute graft dysfunction without biopsy confirmation. Biopsy-proven acute rejection episodes (BPAR) were classified as cellular or antibody-mediated rejection episodes (AMR). Renal function was estimated by glomerular filtration rate (GFR) using the chronic kidney disease epidemiology study (CKD-Epi) equation^21^.

### Outcomes

The primary outcome was composed of death, graft loss, and 1-year graft function <30 mL/min/1.73m^2^ for the survivors. Given that the study included only standard donors, and the follow-up period was limited to one year post-transplantation, a low graft function was considered for composing the primary outcome. The 30 mL/min/1.73m^2^ GFR was chosen because this defines patients as having CKD stage 4, the pre-dialysis stage, according to Kidney Disease Improving Global Outcomes (KDIGO) classification^22^. The secondary outcomes were delayed graft function (DGF), CMV-related events (considering infection and disease), AR, and 1-year graft function.

### Statistical Analysis

The continuous variables were evaluated by the Kolmogorov-Smirnov normality test and then expressed as median and interquartile ranges (1^st^ and 3^rd^ IQR), as all of them presented a non-normal distribution. Categorical variables were expressed as frequency and percentage. The KDPI variable was analyzed throughout the continuous result, and the recipients were grouped according to four strata: 0–35%, >35%–50%, >50%–85%, and >85%^16^. For the univariate analysis, the patients were stratified according to the occurrence of the primary outcome, and then the continuous variables were compared using the Mann-Whitney U test and the categorical variables using *X* ^2^ or Fisher exact tests. To investigate the variables associated with the primary outcome, variables with a p-value < 0.10 in the univariate analysis were considered for multivariate logistic regression.

The secondary outcomes DGF, AR, and CMV- related events were compared according to the KDPI strata using *X* ^2^. Furthermore, for 1-year graft function, the results across KDPI strata were compared using the Kruskal-Wallis test, adjusted with Bonferroni test. For that, the graft function was imputed by the last observed carried forward (LOCF) approach for patients who had graft loss or died. Graft loss was considered in patients who returned to dialysis. Graft and patient’s survival were estimated by Kaplan-Meier test and compared between KDPI strata using the log-rank test.

Statistical analyses were performed using Statistical Package for the Social Sciences (SPSS, version 29; IBM, Armonk, NY, USA) and statistical significance was defined as p < 0.05, with a 95% confidence interval.

## Results

### Baseline Characteristics of Recipients and Donors and KDPI Disposition

Between 2013 and 2017, 3,059 patients received a kidney from deceased donors, and among these donors, 1,943 (63.5%) were SCD.

Table 1 details the baseline characteristics of the recipients. They had a mean age of 48.5 years, most were female (59.6%) and Afro-Brazilian (55.2%). The predominant cause of CKD was undetermined, accounting for 45.5% of cases, followed by glomerular disease at 17.7%, and diabetes at 16.4% of the cases. The great majority of the recipients, 89.9%, had undergone hemodialysis, with a median duration of 3.4 years from the initiation of dialysis to transplantation. Regarding pre-transplant sensitization, retransplant recipients constituted 9.3% of the cohort, 26.2% had a class I cPRA >0%, and 12.3% had a class II cPRA >0%. The median cold ischemia time was 23 hours. Induction with thymoglobulin was used in 98% of KTRs and maintenance immunosuppression included tacrolimus and prednisone for all patients, combined with azathioprine in 49.2%, MPA in 38.1%, and mTORi in 12.5% of the cases.

**Table 1:** Baseline characteristics of kidney transplant recipients (total and stratified by the primary outcome)

Variables	Results N = 1,943	Primary outcome		p-value
		Yes (n = 280)	No (n = 1,663)	
Age, years	48.5 (37.9-57.6)	50.0 (39.3-59.9)	48.3 (37.6-574)	0.01
Female, n (%)	1,158 (59.6)	146 (52.1)	1,012 (60.9)	0.006
Afro-Brazilian, n (%)	1,072 (55.2)	128 (45.7)	944 (56.8)	0.001
CKD etiology, n (%)				0.17
*Undetermined*	*884 (45.5)*	*123 (43.9)*	*761 (45.8)*	
*Glomerular disease*	*344 (177)*	*64 (22.9)*	*280 (16.8)*	
*Diabetes Mellitus*	*319 (16.4)*	*44 (15.7)*	*275 (16.5)*	
*ADPKD*	*155 (8.0)*	*21 (7.5)*	*134 (8.1)*	
*Urological/CAKUT*	*185 (5.9)*	*25 (9.0)*	*160 (9.6)*	
*Arterial hypertension*	*56 (2.9)*	*3 (1.1)*	*53 (3.2)*	
IgG + CMV serology, n (%)	1,838 (94.6)	264 (94.3)	1,574 (94.6)	0.80
RRT time, years	3.4 (1.9-6.1)	3.7 (2.1-6.4)	3.3 (1.8–6.0)	0.06
HD as RRT type, n (%)	1,746 (89.9)	244 (871)	1,502 (90.3)	0.01
Retransplant – n (%)	180 (9.3)	26 (9.3)	154 (9.3)	0.99
0 MM DR	1,642 (84.5)	229 (81.8)	1,413 (85.0)	0.17
0 MM ABDR – n (%)	141 (7.3)	19 (6.8)	122 (7.3)	0.74
cPRA CI > 0% – n (%)	510 (26.2)	79 (28.2)	431 (25.9)	0.42
cPRA CI 0-50% – n (%)	1,761 (90.6)	247 (88.2)	1,514 (91.0)	0.133
cPRA CI > 50% – n (%)	182 (9.4)	33 (11.8)	149 (9.0)	0.133
cPRA CII > 0% – n (%)	239 (12.3)	37 (13.2)	202 (12.1)	0.61
cPRA CII 0-50% – n (%)	1,829 (94.1)	262 (93.6)	1,567 (94.2)	0.666
cPRA CII > 50% – n (%)	114 (5.9)	18 (6.4)	96 (5.8)	0.666
Thymoglobulin induction, n (%)	1,904 (98)	275 (98.2)	1,629 (98.0)	0.95
Maintenance regimen, n (%)				0.14
*Pred + TAC + AZA*	*955 (49.2)*	*125 (44.6)*	*830 (49.9)*	
*Pred + TAC + MPA*	*741 (38.1)*	*124 (44.3)*	*617 (371)*	
*Pred + TAC + mTORi*	*242 (12.5)*	*30 (10.7)*	*212 (12.7)*	
*Other*	*5 (0.3)*	*1 (0.4)*	*4 (0.2)*	

Abbreviations – AZA, azathioprine; ADPKD, autosomal dominant polycystic kidney disease; CAKUT, congenital anomalies of the kidneys and urinary tracts; CKD, chronic kidney disease; CMV, Cytomegalovirus; cPRA, calculated panel reactive antibody; HD, hemodialysis; MM, Mismatches; MPA, mycophenolate acid; mTORi, mammalian target of rapamycin inhibitors; Pred, prednisone; RRT, renal replacement therapy.

Notes – For continuous variables, the Mann-Whitney U test was used to evaluate differences, while for categorical variables, either the Chi-square test or Fisher’s exact test were employed, as detailed in the Methods section. There were no missing data for all variables considered for the baseline characteristics.

**Figure 1:**
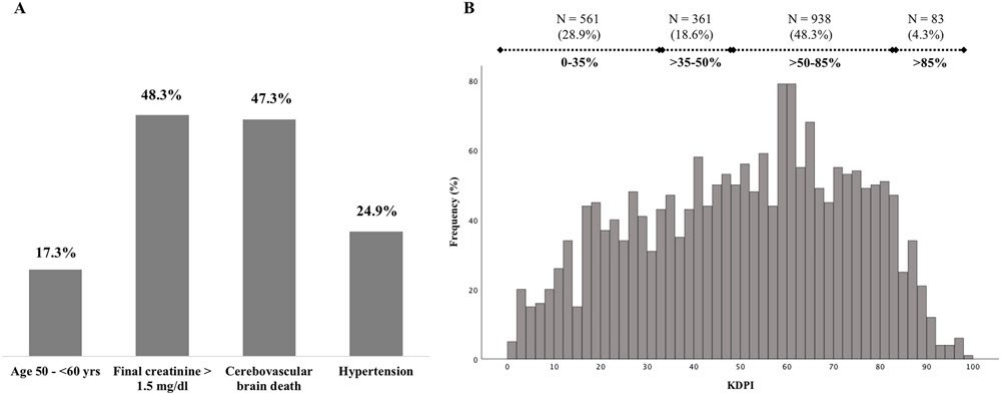
Characteristic that distinguishes standard from extended criteria donors (A) and the KDPI frequency histogram (B).

The donors had a median age of 41.0 years, with 337 of them (17.3%) aged between 50 and 59 years; 61.6% were female and 47.8% Afro-Brazilian. The prevalence of hypertension and diabetes in the donors was 24.9% and 3.8%, respectively. Cerebrovascular injury was the leading cause of brain death, accounting for 47.3% of cases, followed by traumatic injury at 41.2%. The median final creatinine was 1.4 mg/dL, with 938 donors (48.3%) having a final creatinine above 1.5 mg/dL. During the study period (2013–2017), all kidneys were preserved with static solutions prior to implantation, with no difference in the proportion of use concerning the primary outcome. During this period, the most used solution was Eurocollins (56.2%), followed by histidine-tryptophan-ketoglutarate (HTK) solution (30.1%). However, there was a change over the 5 years, with a reduction in the use of Eurocollins from 87.3% to 39.0%, an increase in the use of HTK from 5.2% to 53.2%, while other solutions (7.6% to 7.8%) were maintained. Table 2 summarizes donor characteristics, while Figure 1A highlights the frequency of variables that constitute the UNOS criteria for distinguishing SCD from ECD. The median values of KDRI and KDPI were 1.02 (0.84–1.20) and 52% (32–69), respectively. Only 14 donors had a KDRI higher than 1.7, while more than 50% had a KDPI higher than 50%, with 4.3% higher than 85%. The KDPI frequency histogram and the frequency of each KDPI stratum are depicted in Figure 1B.

**Table 2:** Donors baseline characteristics (total and stratified by the primary outcome in recipients)

Variables	Results N = 1,943	Primary outcome		p-value
		Yes (n = 280)	No (n = 1,663)	
Age, years	41.0 (30.0–48.0)	44.0 (38.0–49.0)	40.0 (30.0–47.0)	< 0.001
Female, n (%)	1,196 (61.6)	161 (575)	1,035 (62.2)	0.132
Afro-Brazilian, n (%)	929 (478)	125 (44.6)	804 (48.3)	0.25
Hypertension, n (%)	484 (24.9)	107 (38.2)	377 (22.7)	< 0.001
Diabetes Mellitus, n (%)	73 (3.8)	16 (5.7)	57 (3.4)	0.063
IgG + CMV serology, n (%)	1,710 (88.0)	253 (90.4)	1,457 (87.6)	0.191
Final creatinine, mg/dL	1.4 (0.9–2.6)	1.5 (1.0–2.8)	1.4 (0.9–2.47)	0.306
Cause of Brain Death, n (%)				
*Cerebrovascular*	*920 (473)*	*153 (54.6)*	*767 (46.1)*	0.151
*Trauma*	*801 (41.2)*	*96 (34.3)*	*705 (42.4)*	
*Anoxia*	*144 (7.4)*	*19 (6.8)*	*125 (7.5)*	
*Other*	*78 (4.0)*	*12 (4.2)*	*66 (3.9)*	
KDRI, index	1.02 (0.84–1.20)	1.11 (0.94–1.30)	1.01 (0.83–1.19)	< 0.001
KDPI, median of %	52.0 (32.0–69–.0)	61 (43.2–76.0)	51.0 (31.0–67.0)	< 0.001
Preservation solution, n (%)				
*Eurocollins*	*1061 (56.2)*	*152 (55.7)*	*909 (56.3)*	0.142
*HTK*	*568 (30.1)*	*74 (27.1)*	*494 (30.6)*	
*Others*	*258 (13.7)*	*47 (172)*	*211 (13.1)*	

Abbreviations – CMV, cytomegalovirus; IgG, immunoglobulin G; KDPI, Kidney Donor Profile Index; KDRI, Kidney Donor Risk Index.

Notes – For continuous variables, the Mann-Whitney U test was used to evaluate differences, while for categorical variables, either the Chi-square test or Fisher’s exact test were employed, as detailed in the Methods section. There were no missing data for all variables considered in baseline characteristics.

### Primary Outcome

The primary outcome occurred in 14.4% of the cases, which included graft loss (4.4%), death (2.9%), and a 1-year eGFR less than 30 mL/min/1.73m^2^ (7.7%). According to the baseline characteristics stratified in Tables 1 (for KTRs) and 2 (for donors), KTRs who experienced the primary outcome were older (p = 0.015), predominantly male (p = 0.006), and white (p = 0.001). Correspondingly, their donors were also older (p < 0.001) and more frequently had a history of hypertension (p < 0.001). The KDRI and KDPI scores were higher in donors whose KTRs experienced the primary outcome: KDRI was 1.11 (0.94–1.30) vs. 1.01 (0.83–1.19), p < 0.001 and KDPI was 61.0% (43.2–76.0) vs. 51.0% (31.0–67.0), p < 0.001. Of note, the frequency of the primary outcome increased with each successive KDPI stratum (Table 3): 8.7% in 0–35%, 13.0% in >35–50%, 17.9% in >50–85%, and 19.3% in >85% (p < 0.001).

**Table 3:** Frequency of primary and secondary outcomes stratified by KDPI

Outcomes	Total N = 1,943	KDPI strata				p-value
		0–35% N = 561	>35–50% N = 361	>50–85% N = 938	> 85% N = 83	
Primary outcome, n (%)	280 (14.4)	49 (8.7)	47 (13.0)	168 (17.9)	16 (19.3)	< 0.001
Secondary outcomes, n (%)						
*DGF, n (%)*	*1,138 (58.6)*	*288 (50.6)*	*214 (59.3)*	*588 (62.7)*	*52 (62.7)*	*< 0.001*
*CMV-related events, n (%)*	*737 (379)*	*206 (36.7)*	*132 (36.6)*	*362 (38.6)*	*37 (44.6)*	*0.50*
*AR, n (%)*	*350 (18.0)*	*100 (17.8)*	*55 (15.2)*	*171 (18.2)*	*24 (28.9)*	*0.03*
*Presumed AR, n (%)*	*24 (1.2)*	*10 (1.8)*	*3 (0.8)*	*11 (1.2)*	*–*	*0.40*
*BPAR, n (%)*	*326 (16.8)*	*90 (16.0)*	*52 (14.4)*	*160 (171)*	*24 (28.5)*	*0.01*
*Cellular AR, n (%)*	*320 (16.5)*	*88 (15.7)*	*51 (14.1)*	*157 (16.7)*	*24 (28.9)*	*0.08*
*AMR, n (%)*	6 (0.3)*	*2 (0.4)*	*1 (0.3)*	*3 (0.3)*	*3 (0.3)*	

Abbreviations – AR, acute rejection; AMR, antibody-mediated rejection; BPAR, biopsy-proven acute rejection; CMV, Cytomegalovirus; CR, Creatinine; DGF, delayed graft function.

Notes – Presumed acute rejection was defined as a successfully treated episode of acute graft dysfunction without biopsy confirmation. *One patient who presented mixed cellular and antibody-mediated rejection was accounted in the AMR category. There were no missing data.

The logistic regression analysis (Table 4) showed that the primary outcome was associated with longer dialysis duration before transplantation (RR for each year = 1.03; 95% CI = 1.00–1.06; p = 0.04), CMV-related events (RR yes vs. no = 1.33; 95% CI = 1.02–1.73; p = 0.03), and AR (RR yes vs. no = 2.12; 95% CI = 1.58–2.84; p < 0.001), as well as the KDPI strata. Compared with the 1–35% KDPI stratum, the RRs were significantly higher in higher KDPI strata: 1.62 (95% CI = 1.06–2.48; p = 0.03) for >35–50%, 2.28 (95% CI = 1.62–3.20; p < 0.001) for >50–85%, and 2.23 (95% CI = 1.19–4.18; p = 0.01) for >85%.

**Table 4:** Logistic regression for the primary outcome

Variables	RR	95% CI	p
Time in dialysis (for each year)	1.03	1.00–1.06	0.04
CMV-related events (yes vs. no)	1.33	1.02–1.73	0.03
KDPI (1–35% as reference)	–	–	–
>35–50%	1.62	1.06–2.48	0.03
>50–85%	2.28	1.62–3.20	< 0.001
>85%	2.23	1.19–4.18	0.01
AR (yes vs. no)	2.12	1.58–2.84	< 0.001

Abbreviations – AR, acute rejection; CMV, cytomegalovirus; KDPI, Kidney Donor Profile Index.

### Secondary Outcomes

**Figure 2:**
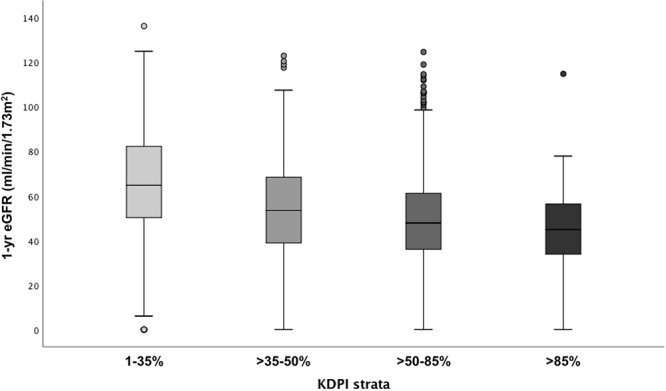
One-year eGFR according to KDPI strata.

The median one-year eGFR across the entire cohort was 52.8 (38.9–68.8) mL/min/1.73m^2^. This was significantly lower in KTRs with donors in higher KDPI strata (p < 0.001): 65.7 (52.4–82.8) in KDPI 1–35%, 55.0 (41.7–69.8) in >35–50%, 49.1 (37.2–62.2) in >50–85%, and 45.1 (35.5–57.6) in KDPI >85% (Figure 2). In a post-hoc pairwise analysis, adjusted with the Bonferroni test, the differences across each KDPI stratum were statistically significant, except between >50–85% and >85% (p = 0.75).

The frequencies of DGF, AR, and CMV-related events were 58.6%, 18%, and 37.9%, respectively, as detailed in Table 3. While the incidence of DGF was higher in KTRs receiving grafts from donors in higher KDPI strata (p < 0.001), there was no significant difference in the incidence of CMV-related events (p = 0.50). However, the incidence of AR was significantly higher in KTRs with donors from the KDPI >85% stratum (p = 0.03).

### Graft and Patient Survival

**Figure 3:**
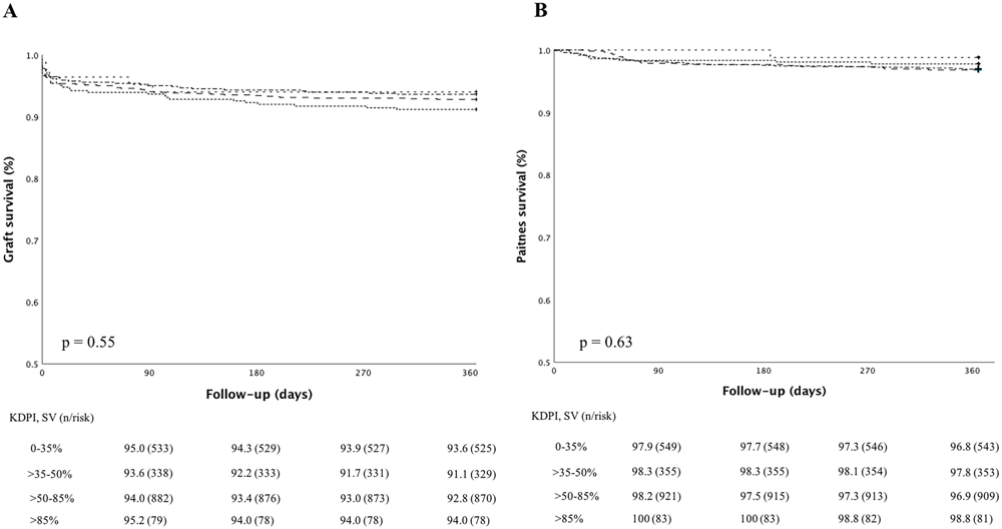
Graft and patient’s survival according to KDPI strata.

There were no differences in non-censured graft survival and patient survival stratified by KDPI. One-year graft survival was 93.6%, 91.1%, 92.8%, and 94.0% according to increasing KDPI strata (p = 0.55) (Figure 3A). Finally, 1-year patient survival was 96.8%, 97.8%, 96.9%, and 98.8% for the increasing KDPI strata (p = 0.63) (Figure 3B).

## Discussion

The shortage of kidneys for transplantation has led to the expansion of criteria for accepting deceased donors, which does not compromise the outcomes of the recipients, compared to remaining on dialysis^23^. Several tools have been developed over time and widely used to better understand the impact of donor characteristics on the recipient clinical outcomes, but they need to be validated in populations different from those included in the studies that originated such tools. The present analysis explored the overlap of the UNOS criteria and the KDPI, the two most useful scoring systems for donor classification, specifically focusing on SCD. As patients were followed-up for only one year post-transplantation, the eGFR composed the outcome as a surrogate for long-term graft survival. Of note, even among SCD recipients, a higher KDPI was associated with an increased probability of the primary outcome (composed of death, graft loss, and 1-year eGFR) and with a 1-year lower eGFR. Considering demographic peculiarities and the structures of health care services, some initiatives to validate the KDRI/KDPI have gained prominence in the last decade^11,12,13,14,15^, but this scale has not been validated in the Brazilian population. The present study is part of a series of analyses aimed at validating the KDPI in the deceased donor population in Brazil. The first analysis has been previously published and included the entire transplanted population with deceased donors at our center between 2013 and 2017, from which this sub-analysis originated. In that analysis, we observed a strong association between KDPI strata and DGF, CMV-related events, and the recipients’ 1-yr-eGFR^16^. In the following analysis, we will investigate the interaction between KDPI and DGF across the entire cohort, not just within SCD cases. Additionally, we have updated the follow-up for long-term outcomes and assessed predictive performance using measures such as discrimination and calibration.

Classically, the UNOS criteria identifies donor characteristics that increase the risk of long-term graft loss by 70%, termed ECD. The study reflected the demographic characteristics of donors in the USA in the 1990s, and 15% of them were classified in the ECD^8^. Notably, applying the UNOS criteria leads to better outcomes for SCD recipients, even if in some cases satisfactory early renal function is not achieved. With the aim of finding a better association between donor-derived scores and recipient outcomes, the KDRI/KDPI emerged a decade later with a more granular analysis of risk^9^. By incorporating other variables and offering a more granular scale, the new score improved donor comparison by facilitating more comprehensive evaluations. Although ECD represented only 15% of the UNOS derivative cohort, one-third of donors falling below the 85^th^ percentile was categorized as ECD, and almost 5% of those above the 85^th^ percentile was classified as SCD^9^. Interestingly, even with demographic changes of the last 20 years and a population with quite different characteristics from American donors, we also found that 5% of the ECD were above the 85^th^ KDPI percentile.

In the present study we observed that risk-defining variables such as cerebrovascular death and alteration in final creatinine were present in about half of SCD. In the study that the UNOS criteria derived from, 39% of the kidneys were from donors with cerebrovascular death, and among them, 52% were under 50 years of age^8^. Among SCD donors, 47.3% had cerebrovascular death. Although fatal cerebrovascular events are on a decreasing trend in high-income countries, over the last three decades, stroke remains the leading cause of death in Brazil, causing more than 100,000 fatal events per year^24,25^. Another epidemiological change that is probably associated with this finding is the alarming increase in cerebrovascular events in individuals under 55 years old worldwide^26,27^.

For the risk calculation in the KDPI/KDRI derivation, a creatinine value of 1.0 mg/dL was assumed for the reference donor^9^. Although creatinine was a linear variable in the initial mathematical model, a weight adjustment was required when the donor’s creatinine was greater than 1.5 mg/dL. Another important characteristic among SCD in our study was the high frequency of those with elevated creatinine, 48.2%, in contrast to 12% in the study for the UNOS criteria^8^. On the other hand, final creatinine is a variable that must be carefully evaluated, as it does not necessarily reflect the donor’s baseline renal function, but rather the acute injuries associated with the process of brain death, and in our setting, the difficulties associated with health services notifying potential donors^28^. For instance, in a Brazilian multicenter study designed to assess the incidence and variables associated with DGF, which included 4,156 transplants from deceased donors between 2014 and 2015 (50% of the country’s transplant activity), the donors’ median serum creatinine was 1.3 mg/dL, and more than a quarter had values greater than 2.0 mg/dL^29,30^. However, a quarter of SCD had hypertension, and 17% were older than 50 years, underscoring that Brazilian SCD is not so standard.

As mentioned above, some initiatives have validated the KDRI/KDPI in different populations in the last decade^11,12,13,14,15^. One of the earliest studies that critically analyzed the index derived from the American population was an analysis based on the UK Transplant Registry. The study included data from 7,620 donors and observed that traditional variables such as age and hypertension were significantly associated with outcomes up to 3 years post-transplant^12^. Notably, they observed that other factors such as body weight, length of hospital stay before death, and the type of vasoactive drug used were also associated with outcomes. The US and UK scores achieved a C-statistic for death-non-censored graft survival of 0.63 and 0.59, respectively. More recently, a European study assessing kidney transplant recipients from a single center in Germany observed a significant association between the KDPI and recipients’ 1-yr-eGFR^13^. The range of KDPI was also associated with the likelihood of graft survival up to 10 years of follow-up.

Besides demonstrating the need for validation and calibration of the KDPI in different populations, the results indicate that kidney function at one year predicts long-term outcomes in performance evaluation analyses of the KDRI/KDPI^15^. Although we did not observe a difference in graft survival in recipients of SCD one year after transplant according to the KDPI strata, there was an inverse association between the KDPI strata and recipient 1-yr-eGFR, similar to what was observed when the whole cohort was included^16^. This result explains the progressive increase in the frequency of the primary outcome with increasing KDPI strata.

Even evaluating a population of recipients from potentially better donors (SCD) and including events traditionally associated with worse prognosis, such as acute rejection and CMV infection, the KDPI stratum was independently associated with the primary outcome in our study. Despite the original American cohort achieving a C-statistic of 0.62 for death-non-censored graft survival, at KDPI extremes, the score performance increased to 0.78^9^. Even among the SCD, risk variables such as age are not uniformly distributed. Our cohort observed a significant median difference of 10% in donor age between recipients who did and did not achieve the primary outcome. A previous study evaluating only adult donors observed a strong correlation between age and KDPI (R^2^ = 0.85), demonstrating that the increments in KDPI were predominantly caused by an increase in donor age^15^.

Our study has some limitations such as the retrospective design, single-center data source, recipient management, including immunosuppressive regimens and CMV reducing risk strategy, and short follow-up time. Despite the data being from a single center, our center performs more than 15% of all transplants in Brazil and might represent the main demographic characteristic of recipient and donors. Moreover, the risk of retrospective bias is reduced by the absence of missing data among all variables of interest. On the other hand, considering the peculiar characteristics of the Brazilian transplantation program, we showed that, even among SCD recipients, KDPI was independently associated with the primary outcome, as well as with DGF and 1-yr-AR incidence.

## Data Availability

The data that support the findings of this study are available from the corresponding author upon reasonable request.
