# Supplementary material for: The association between the Kidney Donor Profile Index and one-year outcomes in Brazilian kidney transplant recipients of standard criteria donors
Source: J Bras Nefrol. 2025 Jul 21;47(3):e20240219. doi: 10.1590/2175-8239-JBN-2024-0219en (PMC12282608; doi:10.1590/2175-8239-JBN-2024-0219en)
Supplement: Translation

# Translation: Associação entre o *Kidney Donor Profile Index e* os desfechos em um ano em receptores de transplante renal com doadores de critério padrão no Brasil

**DOI:** 10.1590/2175-8239-JBN-2024-0219pt

**Authors:** Ana Paula Aquino de Morais, Renato Demarchi Foresto, Maria Amélia Aguiar Hazin, Bianca Cristina Cassão, Helio Tedesco-Silva, José Medina Pestana, Lúcio Requião-Moura

**Affiliations:** 1 Universidade Federal de São Paulo, Departamento de Medicina, São Paulo, SP, Brasil.; 2 Fundação Oswaldo Ramos, Hospital do Rim, São Paulo, SP, Brasil.

**Keywords:** Transplante de Rim, Doadores de Tecidos, Morte Encefálica, Sobrevida do Enxerto, Taxa de Filtração Glomerular

## Abstract

**Introdução:**

O KDPI não foi previamente validado no Brasil, portanto, nosso objetivo foi investigar a associação entre índice e desfechos em um ano em receptores de transplante renal (RTR) com doadores de critério padrão (DCP).

**Métodos:**

Análise de coorte retrospectiva incluindo 1.943 RTR recebendo rins de DCP entre 2013–2017. O desfecho primário foi óbito, perda do enxerto, função do enxerto em um ano <30 mL/min/1,73m^2^ (TFGe, CKD-EPI). A regressão logística identificou variáveis associadas ao desfecho primário. A TFGe em um ano nos estratos de KDPI foi comparada pelo teste de Kruskal-Wallis com correção de Bonferroni.

**Resultados:**

Os doadores tinham 41,0 anos, 24,9% tinham hipertensão, 47,3% morreram por lesão cerebrovascular, 48,3% apresentaram creatinina final >1,5 mg/dL; KDPI mediano foi 52%. O desfecho primário ocorreu em 14,4% dos casos, associado à maior duração da diálise antes do transplante (p = 0,04), eventos relacionados ao CMV (p = 0,03), rejeição aguda (p < 0,001) e estratos de KDPI. Comparado ao estrato KDPI de 1–35%, os RRs para desfecho primário foram significativamente maiores nos estratos KDPI mais elevados: 1,62 (p = 0,03) para >35–50%, 2,28 (p < 0,001) para >50–85%, 2,23 (p = 0,01) para >85%. TFGe em um ano também foi significativamente menor nos RTR com doadores em estratos KDPI maiores (p < 0,001).

**Conclusão:**

O KDPI associou-se de forma independente ao desfecho primário composto por óbito, perda do enxerto e TFGe em um ano <30 mL/min/1,73 m^2^ em receptores com DCP. Apesar de não previamente validado para doadores brasileiros, o índice também foi significativamente associado à TFGe em um ano, função retardada do enxerto e rejeição aguda nesses receptores.

## Introdução

A doença renal crônica (DRC) em estágio terminal constitui uma preocupação premente de saúde pública^1,2^, sendo o transplante renal a melhor opção de tratamento de longo prazo para pacientes nessa condição^3,4^. No entanto, mesmo em países com programas de transplante em larga escala, como o Brasil, ainda há uma escassez de doadores de órgãos^5^. Essa escassez de doadores motivou, nas últimas décadas, uma reavaliação dos critérios de aceitação de doadores, levando à inclusão de classificações de risco baseadas nas características do doador em avaliações de desfechos de longo prazo, afetando a decisão sobre a utilização de órgãos que, de outra forma, poderiam ser descartados^6,7^. Portanto, nas últimas duas décadas, os doadores de rim falecidos passaram a ser categorizados em doadores de critério padrão ou expandido (DCP/DCE) com base nos critérios *da United Network for Organ Sharing* (UNOS). Foram identificadas características dos doadores que, isoladamente ou em combinação, representam um risco 70% maior de perda do enxerto em longo prazo, distinguindo entre DCE e DCP^8^.

Embora os critérios da UNOS ofereçam orientações valiosas às equipes de transplante, eles apresentam limitações como a não consideração do diabetes como um fator prognóstico relevante, a subestimação do risco associado a doadores jovens com múltiplas comorbidades e a superestimação do risco de doadores idosos previamente saudáveis. Para abordar essas limitações e aprimorar a precisão da avaliação de risco, Rao et al. propuseram o *Kidney Donor Risk Index* (KDRI), que incorpora uma gama mais ampla de variáveis na fórmula de predição^9^. Os resultados do KDRI são posteriormente classificados em uma escala de percentis conhecida como *Kidney Donor Profile Index* (KDPI). Essa abordagem oferece às equipes de transplante e aos pacientes uma avaliação mais detalhada da qualidade do órgão, facilitando comparações entre múltiplos doadores para uma tomada de decisão mais fundamentada^10^. Além disso, embora o KDRI/KDPI tenha sido desenvolvido como uma ferramenta de predição de desfechos, seu poder discriminatório é moderado, sendo mais eficaz em casos com níveis de risco extremos^9^.

Embora o KDPI tenha sido validado em alguns países^11,12,13,14,15^, essa validação ainda não ocorreu no Brasil. Apesar das investigações iniciais sobre a sobreposição dos dois sistemas de pontuação durante a criação do KDRI, são necessárias pesquisas adicionais em populações de diferentes países. Notavelmente, na coorte utilizada para estabelecer os critérios da UNOS, aproximadamente 15% dos doadores atendiam à classificação de DCE^8^. No entanto, na coorte a partir da qual o KDRI foi derivado, 32,8% dos doadores que ficaram abaixo do percentil 85 foram classificados como DCE, e 4,6% que excederam o percentil 85 foram classificados como DCP^9^. Essa divergência é crucial, considerando-se as melhorias esperadas nos desfechos para receptores de órgãos de DCP.

Considerando a sobreposição entre o KDPI e os critérios da UNOS e que, em nossa análise anterior, todos os doadores com KDPI <50% eram DCP, mas ainda assim 72,8% e 9,8% dos doadores foram classificados como DCP nos estratos KDPI 51–85% e >85%, respectivamente,^16^ levantamos a hipótese de que mesmo os receptores de rins considerados padrão, mas com KDPI mais elevado, apresentam risco de desfechos desfavoráveis após o transplante. Dessa forma, este estudo teve como objetivo investigar a associação entre o KDPI e os desfechos em um ano em receptores de transplante renal (RTR) de DCP em uma população para a qual o índice não foi previamente validado, como uma das etapas para a validação do KDPI para doadores brasileiros.

## Métodos

### Desenho do Estudo e População

Este foi um estudo de coorte retrospectivo de centro único realizado no Hospital do Rim, em São Paulo, SP, Brasil. Os RTR adultos transplantados entre janeiro de 2013 e dezembro de 2017 e que receberam um enxerto renal de DCP foram incluídos e acompanhados por 12 meses após o transplante. O estudo foi conduzido de acordo com a Declaração de Helsinque e aprovado pelo Comitê de Ética local (número de identificação CAEE 10021419.0.0000.5505 e número de aprovação 3.274.985). O termo de consentimento livre e esclarecido foi dispensado. Os autores tiveram acesso aos prontuários médicos eletrônicos e aos bancos de dados institucionais após a aprovação do Comitê de Ética. A coleta e curadoria dos dados foram realizadas entre 2 de maio de 2019 e 21 de janeiro de 2021. O conjunto de dados foi anonimizado e desidentificado. Para a concepção e apresentação do estudo, os autores utilizaram como referência os checklists STROBE e RECORD, conforme publicado anteriormente^17^.

Os participantes elegíveis foram RTR que foram submetidos a transplante renal de doadores falecidos sob qualquer regime de imunossupressão de manutenção. De 2013 a 2017, foram realizados 3.161 transplantes renais de doadores falecidos no Hospital do Rim. Foram excluídos os pacientes que receberam transplante renal combinado com outro órgão sólido (101 rim-pâncreas e 1 rim-fígado) bem como aqueles que receberam um enxerto renal de DCE (n = 1.116). Assim, 1.943 indivíduos foram incluídos na presente análise. Como o objetivo do estudo foi avaliar os desfechos nos receptores, nos casos em que um único doador forneceu ambos os rins para os receptores incluídos na coorte, as características do doador foram consideradas separadamente para cada receptor correspondente.

### Imunossupressão e Definições

Somente os pacientes com anticorpos doador específico (DSA) nos loci HLA-A e B e DR com intensidade média de fluorescência (IMF) inferior a 1.500 receberam transplante renal. Aqueles com DSA nos loci HLA-A e B e DR com IMF entre 300 e 1.500 receberam transplante renal e um adicional de 1 mg/ kg de globulina antitimócito de coelho (r-ATG) após a dose de indução padrão. De acordo com as práticas locais, todos os pacientes recebem terapia de indução com uma dose única de 3,0 mg/kg de timoglobulina^18^. O regime de imunossupressão de manutenção consiste em tacrolimo, prednisona e um terceiro medicamento definido pelo risco imunológico dos receptores, estratificado pelo painel de reatividade de anticorpos (PRAc): ácido micofenólico (MPA) para receptores com PRAc ≥50% e azatioprina ou inibidores da proteína alvo da rapamicina em mamíferos (mTORi) para aqueles com PRAc <50%.

Os pacientes com PRA ≥ 50% recebem MPA na dose de 720 mg duas vezes ao dia. O tacrolimo é inicialmente prescrito na dose de 0,1 mg/kg duas vezes ao dia e posteriormente ajustado para manter um nível de vale entre 5 e 10 ng/mL. Os receptores de rins de DCP com PRA<50% recebem azatioprina 2 mg/kg uma vez ao dia ou mTORi, de acordo com outras características clínicas do receptor. O tacrolimo é prescrito inicialmente em uma dose de 0,1 mg/kg duas vezes ao dia e posteriormente ajustado para manter um nível de vale entre 8 e 12 ng/mL para os pacientes que estão em uso de azatioprina. Para aqueles que recebem mTORi, o tacrolimo é prescrito inicialmente na dose de 0,05 mg/kg duas vezes ao dia e ajustado para manter um nível de vale entre 3 e 5 ng/mL. Todos os pacientes recebem 0,5 mg/kg de prednisona (dose máxima de 30 mg), com redução gradual semanal até 5 mg no 30° dia. O escore KDPI não foi utilizado como variável para a escolha da imunossupressão em nossa instituição. Conforme a decisão da equipe local, outras características dos doadores ou receptores podem ser consideradas na seleção do terceiro medicamento.

O escore KDPI não é considerado para a decisão sobre o regime de imunossupressão de manutenção. Todos os pacientes recebem profilaxia para *Pneumocystis jirovecii* com sulfametoxazol-trimetoprim. A estratégia adotada para reduzir o risco de eventos relacionados ao citomegalovírus (CMV) é o tratamento preemptivo. Segundo as práticas locais, o evento relacionado ao CMV ocorre quando pacientes em tratamento preemptivo necessitam de estratégia antiviral. Detalhes sobre a estratégia local para tratamento preemptivo foram publicados anteriormente^19^. Em resumo, a viremia por CMV foi monitorada entre 2013 e fevereiro de 2017 com o uso da antigenemia pp65 para tratamento preemptivo. Após fevereiro de 2017, o teste de reação em cadeia da polimerase (PCR) foi incorporado, mas, durante o período do estudo, a decisão de iniciar o tratamento antiviral foi baseada nos resultados da antigenemia. A estratégia preemptiva local recomenda o tratamento antiviral quando 10 ou mais células positivas são detectadas em pacientes assintomáticos ou em qualquer paciente que apresente sintomas de infecção por CMV, independentemente do número de células positivas. O tratamento consiste em 5 mg/kg de ganciclovir intravenoso duas vezes ao dia, ajustado à função renal. O monitoramento foi realizado semanalmente durante o tratamento. Um evento relacionado ao CMV foi definido como qualquer instância em que foi necessário tratamento antiviral, de acordo com os critérios locais.

Desde 2013, o centro tem calculado rotineiramente o KDPI/KDRI para cada doador falecido, com os dados sendo incluídos em prontuários médicos e conjuntos de dados institucionais. Para o presente estudo, todos os dados foram extraídos desses registros e verificados novamente. Nos casos em que o KDRI/KDPI não estavam disponíveis (n = 555; 28,6%), os índices foram calculados retrospectivamente utilizando dados dos EUA correspondentes ao período de coleta e curadoria dos dados. Entre 2013 e 2017, os doadores positivos para hepatite C (HCV) não foram elegíveis para alocação no Brasil. Por fim, o sistema brasileiro de alocação não permite a utilização de órgãos após morte cardíaca (DCD, do inglês *Donation after Circulatory Death*). Portanto, todos os doadores do estudo eram HCV-negativos e apresentaram morte encefálica.

A função retardada do enxerto (FRE) ocorreu quando houve necessidade de diálise na primeira semana após o transplante. A rejeição aguda (RA) foi definida por biópsia de acordo com os critérios de Banff^20^ ou por episódios presumidos, definidos por receptores que apresentaram um episódio de disfunção aguda do enxerto tratado com sucesso sem confirmação por biópsia. Os episódios de rejeição comprovada por biópsia (BPAR, na sigla em inglês) foram classificados como episódios celulares ou mediados por anticorpos (AMR, do inglês *antibody-mediated rejection*). A função renal foi estimada pela taxa de filtração glomerular (TFG) utilizando a equação do estudo CKD-Epi (*Chronic Kidney Disease Epidemiology Study*)^21^.

### Desfechos

O desfecho primário foi composto por óbito, perda do enxerto e função do enxerto em um ano <30 mL/ min/1,73m^2^ para os sobreviventes. Considerando que o estudo incluiu apenas doadores padrão e que o período de acompanhamento foi limitado a um ano após o transplante, uma baixa função do enxerto foi considerada para compor o desfecho primário. A TFG de 30 mL/min/1,73m^2^ foi escolhida porque define os pacientes como portadores de DRC estágio 4, o estágio pré-diálise, segundo a classificação do *Kidney Disease Improving Global Outcomes* (KDIGO)^22^. Os desfechos secundários foram função retardada do enxerto (FRE), eventos relacionados ao CMV (considerando infecção e doença), RA e função do enxerto em um ano.

### Análise Estatística

As variáveis contínuas foram avaliadas pelo teste de normalidade de Kolmogorov-Smirnov e, em seguida, expressas como mediana e intervalos interquartis (IIQ – 1° e 3° quartis), uma vez que todas apresentaram distribuição não normal. As variáveis categóricas foram expressas como frequência e porcentagem. A variável KDPI foi analisada ao longo do resultado contínuo e os receptores foram agrupados de acordo com quatro estratos: 0–35%, >35%-50%, >50%–85% e >85%^16^. Para a análise univariada, os pacientes foram estratificados conforme a ocorrência do desfecho primário e, em seguida, as variáveis contínuas foram comparadas utilizando o teste U de Mann-Whitney, enquanto as variáveis categóricas foram comparadas por meio dos testes *X* ^2^ ou exato de Fisher. Para investigar as variáveis associadas ao desfecho primário, foram consideradas para a regressão logística multivariada as variáveis que apresentaram um valor de p < 0,10 na análise univariada.

Os desfechos secundários FRE, RA e eventos relacionados ao CMV foram comparados de acordo com os estratos do KDPI utilizando *X* ^2^. Além disso, para a função do enxerto em 1 ano, os resultados entre os estratos do KDPI foram comparados por meio do teste de Kruskal-Wallis, ajustado com o teste de Bonferroni. Para isso, a função do enxerto foi imputada pela abordagem LOCF (*last observed carried forward*) para pacientes que apresentaram perda do enxerto ou foram a óbito. A perda do enxerto foi considerada em pacientes que retornaram à diálise. A sobrevida do enxerto e do paciente foi estimada pelo teste de Kaplan-Meier e comparada entre os estratos de KDPI por meio do teste log-rank.

As análises estatísticas foram realizadas com o *Statistical Package for the Social Sciences* (SPSS, versão 29; IBM, Armonk, NY, EUA) e a significância estatística foi definida como p < 0,05, com um intervalo de confiança de 95%.

## Resultados

### Características Basais dos Receptores e Doadores e Disposição Do KDPI

Entre 2013 e 2017, 3.059 pacientes receberam rins de doadores falecidos e, entre esses doadores, 1.943 (63,5%) eram DCP.

A Tabela 1 detalha as características basais dos receptores. A média de idade foi de 48,5 anos, a maioria era do sexo feminino (59,6%) e de origem afro-brasileira (55,2%). A causa predominante da DRC foi indeterminada, representando 45,5% dos casos, seguida por doença glomerular, com 17,7%, e diabetes, com 16,4% dos casos. A grande maioria dos receptores, 89,9%, havia sido submetida à hemodiálise, com uma duração mediana de 3,4 anos desde o início da diálise até o transplante. Com relação à sensibilização pré-transplante, os receptores de retransplante constituíam 9,3% da coorte, 26,2% apresentaram cPRA classe I >0% e 12,3% cPRA classe II >0%. O tempo mediano de isquemia fria foi de 23 horas. A indução com timoglobulina foi utilizada em 98% dos RTR e a imunossupressão de manutenção incluiu tacrolimo e prednisona para todos os pacientes, em combinação com azatioprina em 49,2%, MPA em 38,1% e mTORi em 12,5% dos casos.

**Tabela 1:** Características basais dos receptores de transplante renal (total e estratificada pelo desfecho primário)

Variáveis	Resultados N = 1.943	Desfecho primário		Valor de p
		Sim (n = 280)	Não (n = 1.663)	
Idade, anos	48,5 (37,9–57,6)	50,0 (39,3–59,9)	48,3 (37,6–57,4)	0,01
Sexo feminino, n (%)	1.158 (59,6)	146 (52,1)	1.012 (60,9)	0,006
Afro-brasileiros, n (%)	1.072 (55,2)	128 (45,7)	944 (56,8)	0,001
Etiologia da DRC, n (%)				0,17
*Indeterminada*	*884 (45,5)*	*123 (43,9)*	*761 (45,8)*	
*Doença glomerular*	*344 (177)*	*64 (22,9)*	*280 (16,8)*	
*Diabetes Mellitus*	*319 (16,4)*	*44 (15,7)*	*275 (16,5)*	
*DRPAD*	*155 (8,0)*	*21 (7,5)*	*134 (8,1)*	
*Urológica/CAKUT*	*185 (5,9)*	*25 (9,0)*	*160 (9,6)*	
*Hipertensão arterial*	*56 (2,9)*	*3 (1,1)*	*53 (3,2)*	
Sorologia IgG + CMV, n (%)	1.838 (94,6)	264 (94,3)	1.574 (94,6)	0,80
Tempo de TRS, anos	3,4 (1,9–6,1)	3,7 (2,1–6,4)	3,3 (1,8–6,0)	0,06
HD como tipo de TRS, n (%)	1.746 (89,9)	244 (87,1)	1.502 (90,3)	0,01
Retransplante – n (%)	180 (9,3)	26 (9,3)	154 (9,3)	0,99
0 MM DR	1.642 (84,5)	229 (81,8)	1.413 (85,0)	0,17
0 MM ABDR – n (%)	141 (7,3)	19 (6,8)	122 (7,3)	0,74
PRAc CI > 0% – n (%)	510 (26,2)	79 (28,2)	431 (25,9)	0,42
PRAc CI 0–50% – n (%)	1.761 (90,6)	247 (88,2)	1.514 (91,0)	0,133
PRAc CI > 50% – n (%)	182 (9,4)	33 (11,8)	149 (9,0)	0,133
PRAc CII > 0% – n (%)	239 (12,3)	37 (13,2)	202 (12,1)	0,61
PRAc CII 0–50% – n (%)	1.829 (94,1)	262 (93,6)	1.567 (94,2)	0,666
PRAc CII > 50% – n (%)	114 (5.9)	18 (6.4)	96 (5.8)	0,666
Indução de timoglobulina, n (%)	1.904 (98)	275 (98,2)	1.629 (98,0)	0,95
Regime de manutenção, n (%)				0,14
*Pred + TAC + AZA*	*955 (49,2)*	*125 (44,6)*	*830 (49,9)*	
*Pred + TAC + MPA*	*741 (38,1)*	*124 (44,3)*	*617 (371)*	
*Pred + TAC + mTORi*	*242 (12,5)*	*30 (10,7)*	*212 (12,7)*	
*Outros*	*5 (0,3)*	*1 (0,4)*	*4 (0,2)*	

Abreviações – AZA, azatioprina; DRPAD, doença renal policística autossômica dominante; CAKUT, anomalias congênitas dos rins e do trato urinário; DRC, doença renal crônica; CMV, Citomegalovírus; PRAc, painel de reatividade de anticorpos; HD, hemodiálise; MM, Incompatibilidades; MPA, ácido micofenólico; mTORi, inibidores do alvo da rapamicina em mamíferos; Pred, prednisona; TRS, terapia renal substitutiva.

Observações – Para variáveis contínuas, o teste U de Mann-Whitney foi utilizado para avaliar as diferenças, enquanto para variáveis categóricas, foi utilizado o teste qui-quadrado ou o teste exato de Fisher, conforme detalhado na seção Métodos. Não houve dados faltantes para todas as variáveis consideradas nas características basais.

Os doadores apresentaram idade mediana de 41,0 anos, sendo que 337 deles (17,3%) tinham entre 50 e 59 anos; 61,6% eram do sexo feminino e 47,8% eram afro-brasileiros. A prevalência de hipertensão e diabetes entre os doadores foi de 24,9% e 3,8%, respectivamente. A lesão cerebrovascular foi a principal causa de morte encefálica, representando 47,3% dos casos, seguida por lesão traumática, com 41,2%. A mediana da creatinina final foi de 1,4 mg/dL, com 938 doadores (48,3%) apresentando uma creatinina final acima de 1,5 mg/dL. Durante o período do estudo (2013–2017), todos os rins foram preservados com soluções estáticas antes do implante, sem diferença na proporção de utilização em relação ao desfecho primário. Durante esse período, a solução mais utilizada foi a Eurocollins (56,2%), seguida pela solução de histidina-triptofano-cetoglutarato (HTK) (30,1%). No entanto, houve uma mudança ao longo dos 5 anos, com redução no uso de Eurocollins de 87,3% para 39,0%, aumento no uso de HTK de 5,2% para 53,2%, enquanto outras soluções (7,6% para 7,8%) foram mantidas. A Tabela 2 resume as características dos doadores, enquanto a Figura 1A destaca a frequência das variáveis que constituem os critérios da UNOS para distinção entre DCP e DCE. Os valores medianos de KDRI e KDPI foram 1,02 (0,84–1,20) e 52% (32–69), respectivamente. Apenas 14 doadores apresentaram um KDRI superior a 1,7, enquanto mais de 50% apresentaram um KDPI superior a 50%, com 4,3% acima de 85%. O histograma de frequência do KDPI e a frequência de cada estrato do índice estão representados na Figura 1B.

**Tabela 2:** Características basais dos doadores (total e estratificada de acordo com o desfecho primário nos receptores)

Variável	Resultados N = 1.943	Desfecho primário		Valor de p
		Sim (n = 280)	Não (n = 1,663)	
Idade, anos	41,0 (30,0–48,0)	44,0 (38,0–49,0)	40,0 (30,0–47,0)	< 0,001
Sexo feminino, n (%)	1.196 (61,6)	161 (575)	1.035 (62,2)	0,132
Afro-brasileiros, n (%)	929 (47,8)	125 (44,6)	804 (48,3)	0,25
Hipertensão, n (%)	484 (24,9)	107 (38,2)	377 (22,7)	< 0,001
Diabetes Mellitus, n (%)	73 (3,8)	16 (5,7)	57 (3,4)	0,063
Sorologia IgG + CMV, n (%)	1.710 (88,0)	253 (90,4)	1.457 (87,6)	0,191
Creatinina final, mg/dL	1,4 (0,9–2,6)	1,5 (1,0–2,8)	1,4 (0,9–2,47)	0,306
Causa de morte encefálica, n (%)				
*Cerebrovascular*	*920 (473)*	*153 (54,6)*	*767 (46,1)*	0,151
*Trauma*	*801 (41,2)*	*96 (34,3)*	*705 (42,4)*	
*Anóxia*	*144 (7,4)*	*19 (6,8)*	*125 (7,5)*	
*Outras*	*78 (4,0)*	*12 (4,2)*	*66 (3,9)*	
KDRI, índice	1,02 (0,84–1,20)	1,11 (0,94–1,30)	1,01 (0,83–1,19)	< 0,001
KDPI, mediana em %	52,0 (32,0–69,0)	61 (43,2–76,0)	51,0 (31,0–670)	< 0,001
Solução de preservação, n (%)				
*Eurocollins*	*1061 (56,2)*	*152 (55,7)*	*909 (56,3)*	0,142
*HTK*	*568 (30,1)*	*74 (27,1)*	*494 (30,6)*	
*Outras*	*258 (13,7)*	*47 (17,2)*	*211 (13,1)*	

Abreviações – CMV, citomegalovírus; IgG, imunoglobulina G; KDPI, *Kidney Donor Profile Index*; KDRI, *Kidney Donor Risk Index*.

Observações – Para variáveis contínuas, o teste U de Mann-Whitney foi utilizado para avaliar as diferenças, enquanto para variáveis categóricas, foram empregados o teste qui-quadrado ou o teste exato de Fisher, conforme detalhado na seção Métodos. Não houve dados faltantes para todas as variáveis consideradas nas características basais.

**Figura 1:** Característica que distingue doadores de critério padrão dos de critério expandido (A) e histograma de frequência do KDPI (B).

### Desfecho Primário

O desfecho primário ocorreu em 14,4% dos casos, que incluíram perda do enxerto (4,4%), óbito (2,9%) e TFGe em um ano inferior a 30 mL/min/1,73m^2^ (7,7%). De acordo com as características basais estratificadas nas Tabelas 1 (para os RTR) e 2 (para os doadores), os RTR que apresentaram o desfecho primário eram mais velhos (p = 0,015), predominantemente do sexo masculino (p = 0,006) e brancos (p = 0,001). Da mesma forma, seus doadores também eram mais velhos (p < 0,001) e apresentaram com maior frequência histórico de hipertensão (p < 0,001). Os escores KDRI e KDPI foram mais elevados nos doadores cujos RTR apresentaram o desfecho primário: o KDRI foi de 1,11 (0,94–1,30) vs. 1,01 (0,83–1,19), p < 0,001 e o KDPI foi de 61,0% (43,2–76,0) vs. 51,0% (31,0–67,0), p < 0,001. É importante observar que a frequência do desfecho primário aumentou a cada estrato sucessivo de KDPI (Tabela 3): 8,7% em 0–35%, 13,0% em >35–50%, 17,9% em >50–85% e 19,3% em >85% (p < 0,001).

**Tabela 3:** Frequência de desfechos primários e secundários estratificados por KDPI

Desfechos	Total N = 1.943	Estratos KDPI				Valor de p
		0–35% N = 561	>35–50% N = 361	>50–85% N = 938	> 85% N = 83	
Desfecho primário, n (%)	280 (14,4)	49 (8,7)	47 (13,0)	168 (17,9)	16 (19,3)	<0,001
Desfechos secundários, n (%)						
*FRE, n (%)*	*1.138 (58,6)*	*288 (50,6)*	*214 (59,3)*	*588 (62,7)*	*52 (62,7)*	*<0,001*
*Eventos relacionados ao CMV, n (%)*	*737 (379)*	*206 (36,7)*	*132 (36,6)*	*362 (38,6)*	*37 (44,6)*	*0,50*
*RA, n (%)*	*350 (18,0)*	*100 (178)*	*55 (15,2)*	*171 (18,2)*	*24 (28,9)*	*0,03*
*RA presumida, n (%)*	*24 (1,2)*	*10 (1,8)*	*3 (0,8)*	*11 (1,2)*	*–*	*0,40*
*BPAR, n (%)*	*326 (16,8)*	*90 (16,0)*	*52 (14,4)*	*160 (17,1)*	*24 (28,5)*	*0,01*
*RA celular n (%)*	*320 (16,5)*	*88 (15,7)*	*51 (14,1)*	*157 (16,7)*	*24 (28,9)*	*0,08*
*AMR, n (%)*	6 (0,3)*	*2 (0,4)*	*1 (0,3)*	*3 (0,3)*	*3 (0,3)*	

Abreviações – RA, rejeição aguda; AMR, rejeição mediada por anticorpos; BPAR, rejeição aguda comprovada por biópsia; CMV, Citomegalovírus; CR, Creatinina; FRE, função retardada do enxerto.

Observações – A rejeição aguda presumida foi definida como um episódio de disfunção aguda do enxerto tratado com sucesso, sem confirmação por biópsia. *Um paciente que apresentou rejeição celular mista e mediada por anticorpos foi contabilizado na categoria AMR. Não houve dados ausentes.

A análise de regressão logística (Tabela 4) demonstrou que o desfecho primário esteve associado a uma maior duração da diálise antes do transplante (RR para cada ano = 1,03; IC 95% = 1,00–1,06; p = 0,04), à ocorrência de eventos relacionados ao CMV (RR sim vs. não = 1,33; IC 95% = 1,02–1,73; p = 0,03) e à RA (RR sim vs. não = 2,12; IC 95% = 1,58–2,84; p < 0,001), bem como aos estratos do KDPI. Em comparação com o estrato de KDPI de 1–35%, os RRs foram significativamente maiores nos estratos de KDPI mais elevados: 1,62 (IC 95% = 1,06–2,48; p = 0,03) para >35–50%, 2,28 (IC 95% = 1,62–3,20; p < 0,001) para >50–85% e 2,23 (IC 95% = 1,19–4,18; p = 0,01) para >85%.

**Tabela 4:** Regressão logística para o desfecho primário

Variáveis	RR	IC 95%	p
Tempo em diálise (para cada ano)	1,03	1,00–1,06	0,04
Eventos relacionados ao CMV (sim vs. não)	1,33	1,02–1,73	0,03
KDPI (1–35% como referência)	–	–	–
>35–50%	1,62	1,06–2,48	0,03
>50–85%	2,28	1,62–3,20	< 0,001
>85%	2,23	1,19–4,18	0,01
RA (sim vs. não)	2,12	1,58–2,84	< 0,001

Abreviações – RA, rejeição aguda; CMV, citomegalovírus; KDPI, *Kidney Donor Profile Index*.

### Desfechos Secundários

A mediana da TFGe em um ano, para toda a coorte, foi de 52,8 (38,9–68,8) mL/min/1.73m^2^. Esse valor foi significativamente menor entre os RTR com doadores em estratos de KDPI mais elevados (p < 0,001): 65,7 (52,4–82,8) no KDPI 1–35%; 55,0 (41,7–69,8) em >35–50%; 49,1 (37,2–62,2) em >50–85%; e 45,1 (35,5–57,6) no KDPI >85% (Figura 2). Em uma análise *post-hoc* pareada, ajustada pelo teste de Bonferroni, as diferenças em cada estrato de KDPI foram estatisticamente significativas, exceto entre >50–85% e >85% (p = 0,75).

**Figura 2:** TFGe em um ano de acordo com os estratos KDPI.

As frequências de FRE, RA e eventos relacionados ao CMV foram de 58,6%, 18% e 37,9%, respectivamente, conforme detalhado na Tabela 3. Embora a incidência de FRE tenha sido maior nos RTR que receberam enxertos de doadores em estratos de KDPI mais elevados (p < 0,001), não houve diferença significativa na incidência de eventos relacionados ao CMV (p = 0,50). Entretanto, a incidência de RA foi significativamente maior nos RTR com doadores do estrato KDPI >85% (p = 0,03).

### Sobrevida do Enxerto e do Paciente

Não houve diferenças na sobrevida do enxerto não censurada e na sobrevida do paciente estratificadas pelo KDPI. A sobrevida do enxerto em um ano foi de 93,6%, 91,1%, 92,8% e 94,0% de acordo com o aumento dos estratos de KDPI (p = 0,55) (Figura 3A). Por fim, a sobrevida dos pacientes em um ano foi de 96,8%, 97,8%, 96,9% e 98,8% para o aumento dos estratos de KDPI (p = 0,63) (Figura 3B).

**Figura 3:** Sobrevida do enxerto e do paciente de acordo com os estratos KDPI.

## Discussão

A escassez de rins para transplante levou à expansão dos critérios para aceitação de doadores falecidos, o que não compromete os desfechos dos receptores, em comparação com a permanência em diálise^23^. Diversas ferramentas foram desenvolvidas ao longo do tempo e são amplamente utilizadas para entender melhor o impacto das características do doador nos desfechos clínicos do receptor; porém elas precisam ser validadas em populações diferentes daquelas incluídas nos estudos que deram origem a tais ferramentas. A presente análise explorou a sobreposição dos critérios da UNOS e o KDPI, os dois sistemas de pontuação mais úteis para a classificação de doadores, com foco específico em DCP. Como os pacientes foram acompanhados por apenas um ano após o transplante, a TFGe compôs os desfechos como um marcador substituto da sobrevida do enxerto em longo prazo. Vale ressaltar que, mesmo entre os receptores de DCP, um KDPI mais elevado foi associado a uma probabilidade aumentada de ocorrência do desfecho primário (composto por óbito, perda do enxerto e TFGe em um ano) e a uma menor TFGe em um ano. Considerando as peculiaridades demográficas e as estruturas dos serviços de saúde, algumas iniciativas para validação do KDRI/KDPI ganharam destaque na última década^11,12,13,14,15^; no entanto, essa escala não foi validada na população brasileira. O presente estudo integra uma série de análises destinadas a validar o KDPI na população de doadores falecidos no Brasil. A primeira análise, já publicada anteriormente, incluiu toda a população transplantada com doadores falecidos em nosso centro entre 2013 e 2017, da qual se originou esta subanálise. Nessa análise, observamos uma forte associação entre os estratos de KDPI e a FRE, os eventos relacionados ao CMV e a TFGe dos receptores em um ano^16^. Na análise a seguir, investigaremos a interação entre KDPI e FRE em toda a coorte, e não apenas nos casos envolvendo DCP. Além disso, atualizamos o acompanhamento dos desfechos de longo prazo e avaliamos o desempenho preditivo utilizando medidas como discriminação e calibração.

Classicamente, os critérios da UNOS identificam as características do doador que aumentam em 70% o risco de perda do enxerto em longo prazo, denominado DCE. O estudo refletiu as características demográficas dos doadores nos EUA na década de 1990, e 15% deles foram classificados como DCE^8^. Notavelmente, a aplicação dos critérios da UNOS leva a desfechos mais favoráveis para os receptores de DCP, mesmo que, em alguns casos, não se obtenha uma função renal precoce satisfatória. Com o objetivo de encontrar uma melhor associação entre os escores derivados do doador e os desfechos do receptor, o KDRI/KDPI surgiu uma década depois com uma análise mais granular do risco^9^. Ao incorporar outras variáveis e oferecer uma escala mais granular, o novo escore aprimorou a comparação entre doadores, facilitando avaliações mais abrangentes. Embora os DCE representassem apenas 15% da coorte derivada da UNOS, um terço dos doadores que estavam abaixo do percentil 85 foi classificado como DCE e quase 5% dos que estavam acima do percentil 85 foram classificados como DCP^9^. Curiosamente, mesmo com as mudanças demográficas dos últimos 20 anos e uma população com características bastante distintas dos doadores americanos, também constatamos que 5% dos DCE estavam acima do percentil 85 do KDPI.

No presente estudo, observamos que as variáveis definidoras de risco, como morte cerebrovascular e alteração na creatinina final, estavam presentes em aproximadamente metade dos doadores classificados como DCP. No estudo que serviu de base para os critérios da UNOS, 39% dos rins eram provenientes de doadores com morte cerebrovascular e, entre eles, 52% tinham menos de 50 anos de idade^8^. Entre os doadores classificados como DCP, 47,3% tiveram morte cerebrovascular. Embora os eventos cerebrovasculares fatais estejam em tendência decrescente em países de alta renda, nas últimas três décadas, o AVC continua sendo a principal causa de óbito no Brasil, responsável por mais de 100.000 eventos fatais por ano^24,25^. Outra mudança epidemiológica que provavelmente está associada a esse achado é o aumento alarmante de eventos cerebrovasculares em indivíduos com menos de 55 anos de idade em todo o mundo^26,27^.

Para o cálculo de risco na derivação do KDPI/KDRI, assumiu-se um valor de creatinina de 1,0 mg/dL para o doador de referência^9^. Embora a creatinina fosse uma variável linear no modelo matemático inicial, foi necessário um ajuste de peso quando a creatinina do doador era superior a 1,5 mg/dL. Outra característica importante entre os DCP em nosso estudo foi a alta frequência de indivíduos com creatinina elevada, 48,2%, em contraste com 12% no estudo que originou os critérios da UNOS^8^. Por outro lado, a creatinina final é uma variável que deve ser cuidadosamente avaliada, pois não reflete necessariamente a função renal basal do doador, mas sim as injúrias agudas associadas ao processo de morte encefálica e, em nosso contexto, às dificuldades associadas aos serviços de saúde em notificar os possíveis doadores^28^. Por exemplo, em um estudo multicêntrico brasileiro desenhado para avaliar a incidência e as variáveis associadas à FRE, que incluiu 4.156 transplantes com doadores falecidos entre 2014 e 2015 (50% da atividade de transplante do país), a creatinina sérica mediana dos doadores foi de 1,3 mg/dL, e mais de um quarto apresentou valores superiores a 2,0 mg/dL^29,30^. No entanto, um quarto dos doadores classificados como DCP apresentava hipertensão e 17% tinham mais de 50 anos de idade, o que ressalta que os DCP no Brasil não estão tão em conformidade com o padrão.

Conforme mencionado acima, algumas iniciativas validaram o KDRI/KDPI em diferentes populações ao longo da última década^11,12,13,14,15^. Um dos primeiros estudos que analisou criticamente o índice derivado da população norte-americana foi uma análise baseada no Registro de Transplantes do Reino Unido. O estudo incluiu dados de 7.620 doadores e observou que as variáveis tradicionais, como idade e hipertensão, foram significativamente associadas aos desfechos até 3 anos após o transplante^12^. Notavelmente, eles observaram que outros fatores, como peso corporal, tempo de permanência no hospital antes do óbito e o tipo de droga vasoativa utilizada, também foram associados aos desfechos. Os escores dos EUA e do Reino Unido alcançaram uma estatística C de 0,63 e 0,59, respectivamente, para a sobrevida do enxerto não censurada para óbito. Mais recentemente, um estudo europeu que avaliou receptores de transplante renal de um único centro na Alemanha observou uma associação significativa entre o KDPI e a TFGe dos receptores em um ano^13^. A faixa do KDPI também esteve associada à probabilidade de sobrevida do enxerto em até 10 anos de acompanhamento.

Além de demonstrar a necessidade de validação e calibração do KDPI em diferentes populações, os resultados indicam que a função renal em um ano pode predizer desfechos de longo prazo nas análises de avaliação de desempenho do KDRI/KDPI^15^. Embora não tenhamos observado uma diferença na sobrevida do enxerto em receptores de DCP um ano após o transplante, conforme os estratos do KDPI, houve uma associação inversa entre os estratos do KDPI e a TFGe do receptor em um ano, semelhante ao que foi observado quando toda a coorte foi incluída^16^. Esse resultado explica o aumento progressivo na frequência do desfecho primário conforme o aumento dos estratos de KDPI.

Mesmo avaliando uma população de receptores provenientes de doadores potencialmente melhores (DCP) e incluindo eventos tradicionalmente associados a um pior prognóstico, como rejeição aguda e infecção por CMV, o estrato KDPI foi independentemente associado ao desfecho primário em nosso estudo. Apesar de a coorte original americana ter alcançado uma estatística C de 0,62 para a sobrevida do enxerto não censurada por óbito, nos extremos do KDPI, o desempenho do escore aumentou para 0,78^9^. Mesmo entre os DCP, as variáveis de risco, como a idade, não são distribuídas de maneira uniforme. Nossa coorte observou uma diferença mediana significativa de 10% na idade do doador entre os receptores que atingiram e os que não atingiram o desfecho primário. Um estudo anterior que avaliou exclusivamente doadores adultos observou uma forte correlação entre a idade e o KDPI (R^2^ = 0.85), demonstrando que os aumentos no KDPI foram predominantemente causados por um aumento na idade do doador^15^.

Nosso estudo apresenta algumas limitações, como o desenho retrospectivo, a fonte de dados de centro único, o manejo dos receptores incluindo os regimes imunossupressores e a estratégia de redução do risco de CMV – e o curto tempo de acompanhamento. Apesar de os dados serem de um único centro, nosso centro realiza mais de 15% de todos os transplantes no Brasil e pode representar a principal característica demográfica dos receptores e doadores. Além disso, o risco de viés retrospectivo é reduzido pela ausência de dados faltantes entre todas as variáveis de interesse. Por outro lado, considerando as características peculiares do programa brasileiro de transplante, demonstramos que, mesmo entre os receptores de DCP, o KDPI foi independentemente associado ao desfecho primário, bem como à FRE e à incidência de RA em um ano.

## Data Availability

Os dados que suportam os resultados deste estudo estão disponíveis através do autor correspondente mediante solicitação razoável.
